# A unidirectional mapping of ICD-8 to ICD-10 codes, for harmonized longitudinal analysis of diseases

**DOI:** 10.1007/s10654-023-01027-y

**Published:** 2023-08-09

**Authors:** Mette Krogh Pedersen, Robert Eriksson, Roc Reguant, Catherine Collin, Helle Krogh Pedersen, Freja Karuna Hemmingsen Sørup, Christian Simon, Anna Marie Birch, Michael Larsen, Anna Pors Nielsen, Kirstine Belling, Søren Brunak

**Affiliations:** 1 Novo Nordisk Foundation Center for Protein Research, University of Copenhagen, 2200 Copenhagen, Denmark; 2 Department of Infectious Diseases, Karolinska University Hospital, Stockholm, Sweden; 3 Division of Infectious Diseases, Department of Medicine, Karolinska Institutet, Stockholm, Sweden; 4 Australian E-Health Research Centre, Commonwealth Scientific and Industrial Research Organisation, Sydney, Australia; 5 Department of Obstetrics and Gynaecology, Holbæk Hospital, Holbæk, Denmark; 6 Department of Ophthalmology, Rigshospitalet-Glostrup, Copenhagen University Hospital, Glostrup, Denmark

**Keywords:** International classification of diseases, ICD-8, ICD-10, Denmark, Disease codes, Diagnosis, Mapping, Conversion table, Crosswalk, Data harmonization

## Abstract

Periodic revisions of the international classification of diseases (ICD) ensure that the classification reflects new practices and knowledge; however, this complicates retrospective research as diagnoses are coded in different versions. For longitudinal disease trajectory studies, a crosswalk is an essential tool and a comprehensive mapping between ICD-8 and ICD-10 has until now been lacking. In this study, we map all ICD-8 morbidity codes to ICD-10 in the expanded Danish ICD version. We mapped ICD-8 codes to ICD-10, using a many-to-one system inspired by general equivalence mappings such that each ICD-8 code maps to a single ICD-10 code. Each ICD-8 code was manually and unidirectionally mapped to a single ICD-10 code based on medical setting and context. Each match was assigned a score (1 of 4 levels) reflecting the quality of the match and, if applicable, a “flag” signalling choices made in the mapping. We provide the first complete mapping of the 8596 ICD-8 morbidity codes to ICD-10 codes. All Danish ICD-8 codes representing diseases were mapped and 5106 (59.4%) achieved the highest consistency score. Only 334 (3.9%) of the ICD-8 codes received the lowest mapping consistency score. The mapping provides a scaffold for translation of ICD-8 to ICD-10, which enable longitudinal disease studies back to and 1969 in Denmark and to 1965 internationally with further adaption.

**Supplementary Information:**

The online version contains supplementary material available at 10.1007/s10654-023-01027-y.

## Introduction

Healthcare data registration transforms individual observations and complex conditions into compact communicable codes. Structured registration of cause of death became compulsory in several countries already during the nineteenth century. In 1948 the World Health Organization (WHO) was created and given the responsibility to further develop and maintain the International Statistical Classification of Diseases and Related Health Problems, often known as the international classification of diseases (ICD). Also in 1948, the WHO published the 6th version of ICD (ICD-6), which incorporated morbidity in addition to causes of mortality. [1] ICD revisions reflect advances in health and medical science, and are essential for continued relevance and accuracy, but complicate data linking across time.

ICD versions 6 through 9 share the same overall structure and differ mainly by increasing granularity. [2] ICD-10, adopted from 1990, not only enlarged the code base, but also regrouped many parts of the classification. [3] The newest version, ICD-11, was preliminary released in 2018 to allow Member States to prepare for implementation, and reporting using ICD-11 commenced in 2022. WHO provides bi-directional mapping, also known as transition tables or crosswalks, between ICD-10 and ICD-11. [4] However, earlier revisions were not accompanied by crosswalks, and the few existing mappings have been created piecemeal to use data from timespans across ICD versions. [5]

The study of electronic health records is a key part of modern medical research—benefits range from improved diagnostics, prognostics and treatment of patients to better health resource allocation. Data harmonization permitting analysis of health care records over long time periods is often necessary for comparison of health status and medical care longitudinally. Research on diseases or procedures generally requires harmonisation, or mapping, of relevant disease and procedure codes to enable comparison of different time periods. Research of data sets covering all diseases in a population, i.e. all registered disease codes, makes it possible to study lifelong and multigenerational disease trajectories, which has led to important results, in some cases providing direct clinical decision-making support. [6–13]

While WHO nomenclature regulations require use the most current ICD revision for morbidity and mortality, many member states create adapted, national versions of the current ICD. National versions may differ from the international version in the level of detail, incomplete adoption of a category, or the addition of e.g. procedure codes. The time period covered by ICD-8 to ICD-10 (from 1965) saw the implementation of disease registration into all clinical practice. A publicly available, direct mapping of ICD-8 to ICD-10 would be valuable to the research community as many datasets coded with ICD-8 are still maintained. [14–16] In countries like Denmark, which did not adopt ICD-9 but transitioned directly from ICD-8 to ICD-10, a mapping from ICD-8 to ICD-10 is obviously of interest. [17] While crosswalks have been created between ICD-9 and ICD-10, [18, 19] the link between ICD-8 and later versions is tenuous.

The ICD-8 to ICD-10 mapping we present here covers the entire ICD including the Danish adaptations to the classification, and it may be used to bridge the gap between the versions. Together with the mapping the civil registration numbers currently allows for analysis of health care data, at high granularity, over more than a half-century.

## Methods

### Datasets

Danish ICD-8 and ICD-10 classifications are available through the Danish Health Data Authority. [14] Supplementary materials on ICD-8 and ICD-10 from WHO and the Danish Health Authority were also used. [21–23]

The Danish National Patient Registry (DNPR) was used for frequencies analysis in Danish coding practice. The DNPR provides nationwide longitudinal administrative and clinical data. It comprises data on all somatic hospital contacts since 1977 and all psychiatric hospital contacts since 1995; recording both primary and secondary ICD diagnoses, and procedures. [24]

### The hierarchical structure of ICD

ICD is hierarchically structured and divides into increasing levels of detail. At the top of the hierarchy are chapters covering disease areas, and at the bottom, thousands of detailed codes. The main chapters and the general hierarchical structure are predominantly preserved throughout revisions. ICD-8 has chapters divided into three-digit codes, most of which subdivide further into four-digit codes. The Nordic countries’ ICD-8 modifications include five-digit codes.

**Fig. 1:**
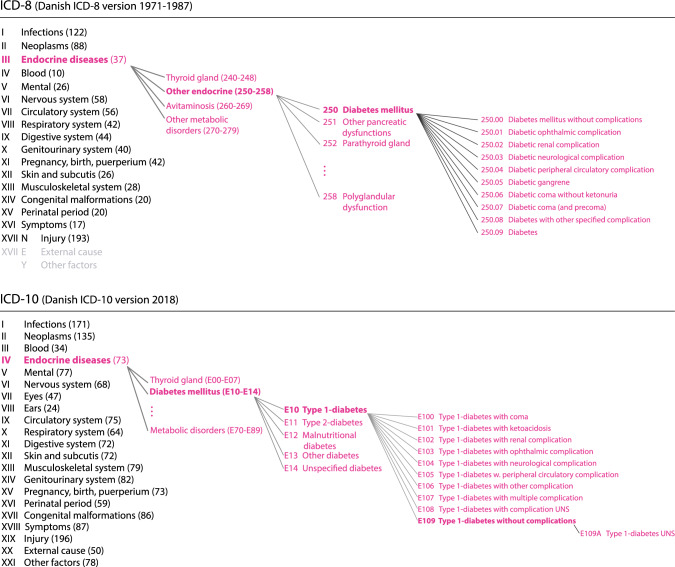
The hierarchical structure of ICD-8 and ICD-10. The structure is illustrated by expanding the endocrine main chapters down to the most detailed diabetes related subcategories. ICD-8 (top) has only a single diabetes category, in contrast, ICD-10 (bottom) categorises different types of diabetes, thus enlarging the diabetes related classification options. The chapter headings are followed by the number of block categories within that chapter. *Chapter names are shortened from the official names to increase illustration readability*

ICD-8 and ICD-10 share part of their overall structure (Fig. 1 and Supplementary Fig. 1). The main changes from ICD-8 to ICD-10, which complicate mapping between them are: (1) the increase in the number of chapters, (2) the restructuring and relocation of codes across chapters, (3) the additional levels of subdivision, (4) the inclusion of new codes, and (5) the change of language, ICD-8 being in Latin and ICD-10 in Danish.

An important point regarding ICD structure is that there is no single principle dictating how diseases subdivide. The subdivision is based on the most suitable medical premise (anatomy, aetiology, pathology, etc.) for each specific case and this varies according to area, hierarchic level, and version of the classification. [14, 25] For example, neoplasms are subdivided first according to behaviour (malignant, benign, or unknown) then according to whether they are primary, secondary or ill-defined, and finally according to anatomic location. Endocrine disorders are subdivided primarily according to anatomy. Infectious diseases are subdivided according to infectious agent. Some key elements are shared by all revisions, e.g. the availability of “Unspecified” and less specific codes, to make sure that all health-related conditions are classifiable, and inclusions and exclusions guide the user to classify correctly. *Incl.* and *excl.* may be placed at all levels in the hierarchical structure.

### The mapping strategy

**Fig. 2:**
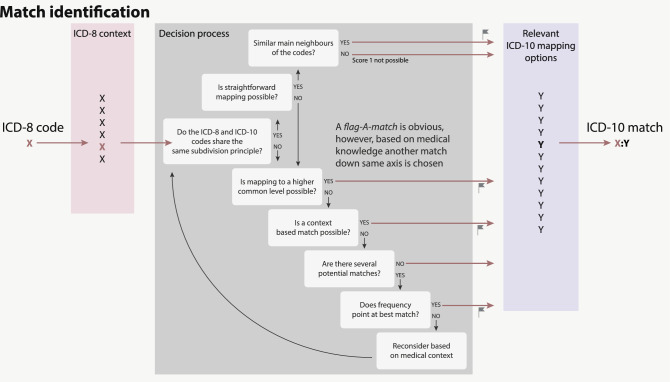
Illustration of the mapping process. The mapping process is based on a careful examination of the ICD-8 and ICD-10 structure (the structure of main chapters, blocks of categories, categories and subcategories). The context of a specific ICD-8 code is taken into consideration when identifying a list of possible ICD-10 matches. If the granularity of the ICD-8 context is similar to the granularity of the ICD-10 options, the mapping is relatively straightforward. If not, mapping to a common higher level is considered. The frequencies of the codes in each ICD revision were used to reflect the actual use as the aim is to combine medical history that span the two terminologies

Working in the Nordic context with Danish data, most importantly the DNPR, we performed the mapping based on the expanded Danish ICD versions. We matched all Danish ICD-8 codes to the Danish ICD-10 version 4.02 as provided by the Danish Health Data Authority. [23] We focused on diseases, excluding codes describing external causes (E-codes) and codes included in*’Other factors’*; classifications regarding concerns other than diseases (Y-codes). The Nordic Medico-Statistical Committee (NOMESCO) have commonly been used in the Nordic countries instead of the WHO ICD-10 chapter XX *‘External causes of morbidity and mortality’*. [26] Danish ICD modifications generally retain the original structure, differing in the language of disease names and in greater granularity. The Danish ICD-8 modification was created in such a fashion that removing the fifth, last digit of a code often resulted in the international code. [21–22] The mapping was performed according to a strategy inspired by the general equivalence mapping database (GEM) [18, 27] and the following premises (Fig. 2):

- The mapping was performed unidirectionally from ICD-8 to ICD-10.
- Each ICD-8 code was translated to one ICD-10 code—not a combination of codes or alternatives. However, several ICD-8 codes could map to the same ICD-10 code. The most suitable mapping was chosen based on medical expertise, assessment of which code choice would be most correct in the research context of analysing past medical records, and frequency of the code in the Danish National Patient Records.
- No information was added, e.g. some ICD-10 codes contain information regarding the method of diagnosis or pathology—which is information not included in the equivalent ICD-8 codes. Despite some information loss, general codes were therefore chosen in these cases, to avoid adding information not present in the ICD-8 codes.
- Mapping was done to the highest granularity possible, to retain the information given by the ICD-8 code.
- Non-diseases in the Danish ICD-8 version (e.g. mortality, accidents, and sexual orientation) were not mapped, see above (Supplementary table 1).

Mapping was done to the highest specificity possible, to retain the information given by the ICD-8 code; while mapping from a less specific to a more specific code was done only as a last resort. Mapping was performed manually by medical doctors, code by code. The full content of a code was defined not only by its name and specifications (such as *incl.* or *excl.*) but also on its position in the hierarchy, including the block name and surrounding codes. Some areas of the Danish ICD-8 and ICD-10 share a common structure and ICD-8 codes therefore mapped directly to ICD-10. In other areas, chapter structure differs and a perfect equal translation at detailed code level was not possible; instead, the ‘best possible match’ was found. Some ICD-8 codes changed title and content between the 1971, 1976 and 1986 Danish editions, and are therefore treated as separate codes with separate ICD-10 matches. We annotated these with the relevant dates.

Psychiatric diagnoses partially follow the “Conversion tables between ICD-8, ICD-9, and ICD-10”, revision 1, provided in 1994 by WHO. [28] The conversion tables are one-to-many and the current mapping has in most cases listed the first example given, as the mapping.

### Data driven decisions in non-trivial cases

In cases of doubt, we conferred with medical specialists about coding practice in Danish hospitals during the relevant time period, and we also analysed code frequencies in the Danish nationwide dataset. Coding practices often develop over time, and knowledge of habitually employed codes within a given specialty is often transferred from doctor to doctor, rather than being implicit in code structure. [29] These data driven matches were based on ICD code frequencies in the nationwide dataset.

### Comments on the mapping: scores and flags

To improve transparency and usability, we assigned all matches a quality score and some matches also a flag, to indicate type of match.

#### Score

The score reflects how similar the two matched codes are in medical content, and how well the ICD-8 code fits into the ICD-10 structure. Essentially, it reflects how much information is retained in the mapping. Scores were assigned manually by the medical doctor performing the mapping.

- *Score 1* A straightforward match covering the exact same structure and information (including specified *incl.*/*excl.* criteria).
- *Score 2* A good match.
- *Score 3* An acceptable match.
- *Score 5***:** The best possible match, where no good match can be made. We use score ‘5’ instead of ‘4’ to emphasize the poor quality of the match.

The following examples illustrate the scoring system (Table 1): ICD-8 code 340.09 “Multiple sclerosis” matches directly to the ICD-10 code G35.9 “Multiple sclerosis unspecified” and the match is a score 1. (‘Unspecified’ is implied in the ICD-8 code). ICD-8 specifies multiple sclerosis by the presentation of clinical symptoms, ICD-10 by phenotype. Consequently, the mapping process loses the information of the clinical manifestations contained in the ICD-8 codes 340.00, 340.01 and 340.08, resulting in the score 3 for these codes. Scoring is an evaluation of how well the ICD-8 code and its ICD-10 match reflect the same clinical condition. Name similarity is not the basis of matches or scores; the meaning of a term depends on the medical content of the term together with its overall context in the ontology. For example: ICD-8 code 572.00 “Pylephlebitis” and ICD-10 code K75.1 “Pylephlebitis” is not a perfect 1:1 mapping due to the finer grained structure of ICD-10. Consequently, this match receives a score of 2, not 1.

**Table 1:** Exemplification of scores and semantic similarity. The hierarchy structure of corresponding ICD-8 and ICD-10 subcategory

ICD-8 (code and title)	ICD-10 match (code)	Score
*340 Multiple sclerosis*		
340.09 Multiple sclerosis (unspecified is implied)	G35.9	1
340.00 Multiple sclerosis with paresis of the urine bladder*	G35	3
340.01 Multiple sclerosis with paresis of extremities*	G35	3
340.08 Multiple sclerosis with other manifestations*	G35	3
*572 Suppurative hepatitis and liver abscess*		
572.09 Suppurative hepatitis and liver abscess (unspecified is implied)	K75.0	3
572.00 Pylephlebitis*	K75.1	2
572.01 Liver abscess*	K75.0	2

ICD-10 (code and title)		
*G35 Multiple sclerosis*		
G35.9 Multiple sclerosis unspecified*		
G35.9A Attack wise multiple sclerosis*		
G35.9B Primary multiple sclerosis *		
G35.9C Progressive multiple sclerosis *		
*K75 Other inflammatory liver disease*		
K75.0 Liver abscess		
K75.0A Haematogenic hepatic abscess*		
K75.0B Lymphogenic hepatic abscess*		
K75.0C Hepatic abscess with cholangitis*		
K75.0D Hepatic abscess with pylephlebitis*		
K75.1 Pylephlebitis …		
K75.9 Inflammatory liver disease, unspecified*		

* Extended codes in the Danish ICD versions. ICD, International Classification of Diseases

#### Flag

Some difficult matches are flagged with one or more of seven flags highlighting the mapping problem, e.g. different subdivision principles in ICD8 and ICD-10 (Supplementary table 2).

## Results

**Fig. 3:**
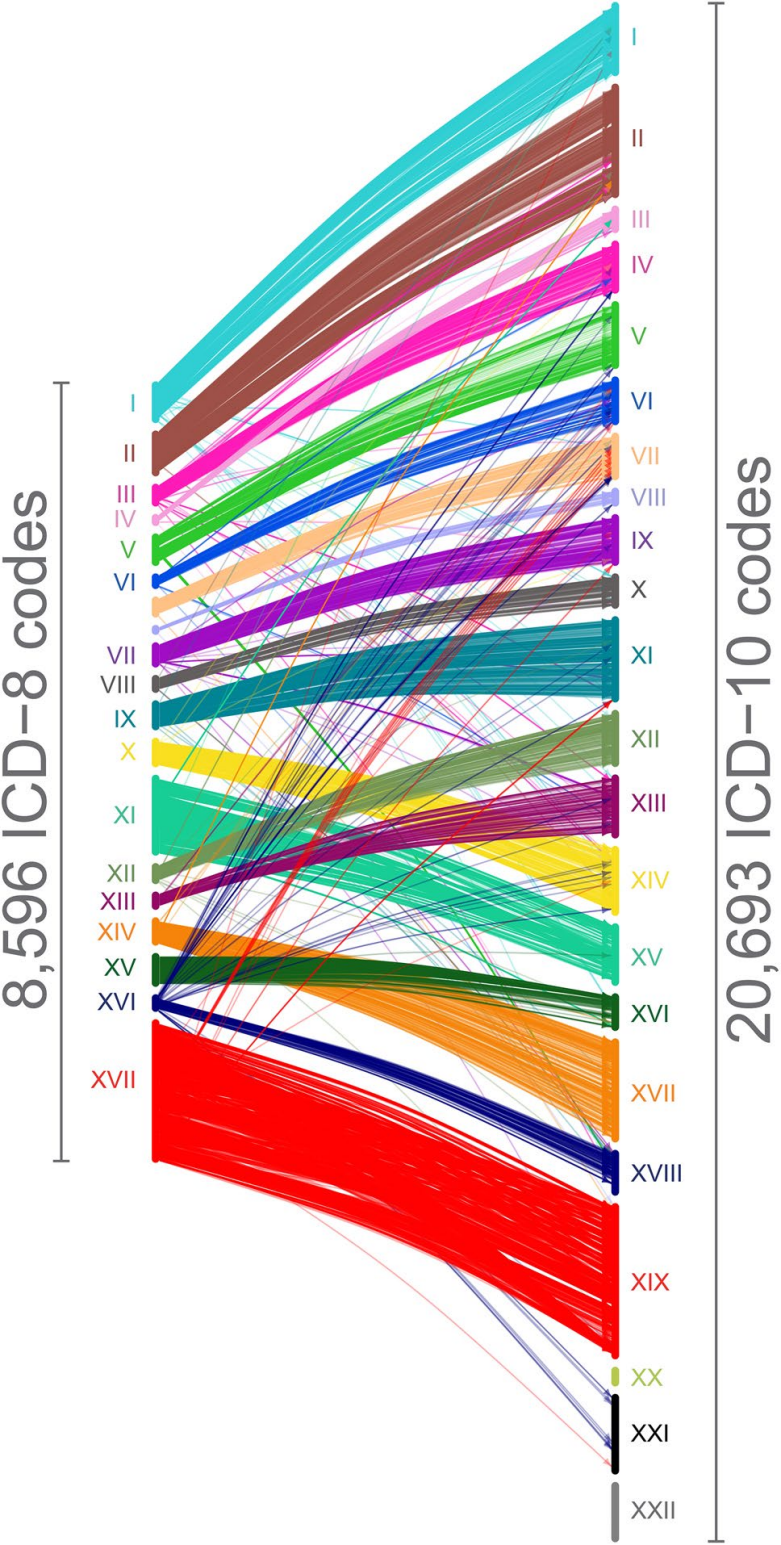
Mapping scores by chapter. The chapters with the best mapping scores are Infectious, Neoplasms, and Congenital malformations. However, the Mental chapter, Pregnancy chapter, the chapter for Conditions originating in the perinatal period, and the chapter for Factors influencing the health status are the chapters with the lowest level of best matches. *Chapter names are shortened from the official names to increase illustration readability*

We manually mapped 8,596 (99.9%) Danish ICD-8 codes out of a total of 8,603 to one of the 20,693 unique codes in the Danish ICD-10 version. The transition table is available for download (Supplementary file 1), together with a technical description (Supplementary table 3). An excellent score of 1 or 2 was achieved by 5106 (59.4%) of all mappings. Only 334 (3.9%) of the ICD-8 codes were so difficult to match that they received the lowest score of 5. In addition, 3484 (40.5%) of the matches were further characterized by one or more of the seven optional flags to aid usability and transparency of the mapping resource (Supplementary table 2). In general, chapters V (Mental disorders), XV (Pregnancy, childbirth and puerperium), XVI (Perinatal period), and XXI (Factors influencing health status) received the lowest scores signalling the perplexity to map these chapters (Fig. 3).

**Fig. 4:**
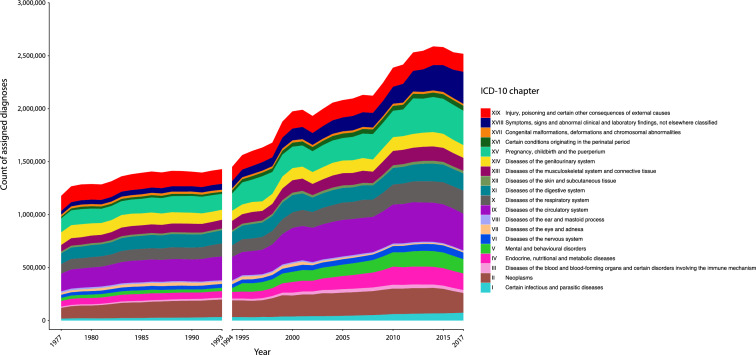
ICD-8 to ICD-10 mapping permits expansion of the timespan for which data sets can be analysed. Annual distribution of diagnoses in the Danish National Patient Registry by ICD-10 chapter shows consistency over the ICD-8 and ICD-10 time periods. The 40-year data span covers both ICD-8 diagnoses (1977–1993, left) and ICD-10 diagnoses (1994–2017, right); ICD-8 diagnoses are collapsed to ICD-10 main chapter categories

**Fig. 5:**
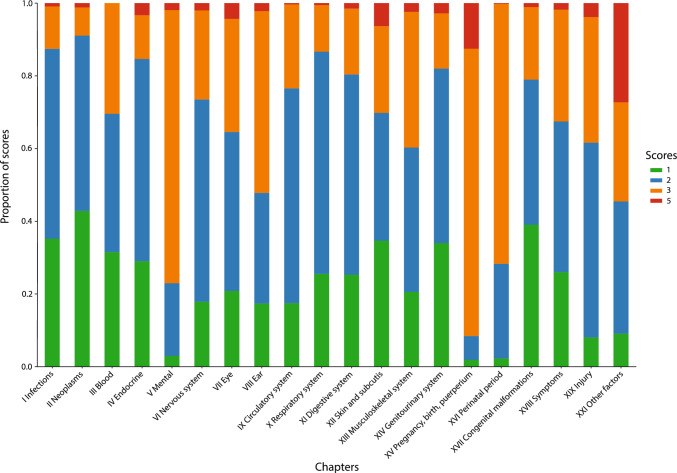
Illustration of the mapping between Danish ICD-8 and ICD-10 codes. A line links each of the 8,596 ICD-8 codes to its match in ICD-10

The ICD-8 to ICD-10 mapping permits consistent analysis of diagnoses in the DNPR over 55 years and expands the number of unique individuals that can be analysed from 7,191,519 to 8,172,534 patients compared with only using ICD-10 codes. The number of ICD-10 diagnoses also increased by 23,495,534 diagnoses reaching a total of 230,320,583 diagnoses. Disease distribution in the ICD-8 and ICD-10 periods is similar, which indicates consistency on the mapped diagnoses (Fig. 4). At the same time, one should notice that medical practices and reporting procedures change so these variations may be affected by external factors and not the mapping. Code-by-code matches of ICD-8 codes to ICD-10 codes show that while there are few exceptions in every chapter, most codes remain in the same chapter across versions (Fig. 5).

## Discussion

With this work, we provide the first complete mapping of all ICD-8 disease codes to ICD-10, enabling the study of disease trajectories as far back as 1965 internationally and 1969 in Denmark. Combined with other resources this mapping enables linkage of ICD-8, ICD-9, ICD-10 and ICD-11 [4]. This makes it possible to harmonize and integrate health care data registered in ICD codes from 1965 to the present.

Many harmonization standards’ usefulness is improved by modification for specific projects and data sets, and this crosswalk is no exception. When using the crosswalk, it is important to consider one’s aim and to check whether adjustments need to be made. Focus on a specific disease area from a specific perspective may require adaptations to reflect this. For example, 311.5 “Mild mental retardation/With chromosomal abnormalities” could correctly be mapped to F709 “Mild mental retardation,” or to Q9, chromosomal abnormalities. For most research purposes, a differentiation between genetic conditions and cognition difficulties due to e.g. neonatal hypoxia will be important, i.e., disease aetiology should be chosen as the coding principle. For example, in a pan-disease trajectory study of morbidity and comorbidities, Q9 is the correct mapping. However, in the context of research into cognitive difficulties, one might consider mapping 311.5 to F70.9 to catch all cases.

Similarly, the code 290.0 “Senile dementia” can be mapped to F00.1 “Dementia in Alzheimer disease with late onset”—which will provide a fairly good representation of how many patients with late onset Alzheimer disease there were in one’s cohort in the years covered by ICD-8, and will allow for comparison across the ICD-8 and ICD-10 period. This is a good example of the principle of mapping to the result with the highest frequency in the National Patient Registry data set. Alternatively, in the case of research specifically into dementia, where precise differentiation is required between certain and uncertain cases of Alzheimer’s, the code “Senile dementia” can be mapped to F03, “Unspecified dementia.” Like all data harmonization and data integration tools, these transition tables will occasionally need to be modified for the research project at hand. The recent literature on mixed-dementia across Alzheimer and vascular dementia adds to this complexity. [30, 31]

This paper is novel in developing a fine-grained quantitative scoring of the qualitative match. In contrast to other published translations, our mapping not only assign matches a binary score. This mapping employs a more informative multi-level scoring system and a flag system.

Periodic revisions of ICD ensure its continuing usability. All levels of the hierarchical structure reflect compromises, considering changes in medical theory and knowledge, clinical practice, requirements and social norms, and the need to be relevant across multiple cultures and medical systems. There is no general consistent hierarchical principle. Besides increasing the number of codes and their granularity, revisions adjust and reorganize whole areas of the classification. Some areas of ICD keep the same structure across revisions while others change, e.g. ICD-8 chapter VI*’Diseases of the nervous system and sensory organs’* splits into three chapters in ICD-10 (Fig. 5). ICD-8 and ICD-10 simply differ too much for a perfect mapping and make a consistent objective standardized transformation impossible. Instead, our matches are compromises based on medical knowledge, clinical practice, and a mapping strategy. The mapping was performed carefully code by code. An essential premise was to create a mapping where one ICD-8 code matches one and only one ICD-10 code in order to enable epidemiological research across ICD revisions.

The transition table described here aims towards a complete mapping of morbidity codes, but the framework could be expanded to translation of mortality codes as some counties transitioned directly from ICD-8 to ICD-10. [32] However, such an effort should be combined with a bridge-coding study.

A more perfect matching is possible through one-to-many relations in mappings as used in GEM and WHO mappings. 'One-to-one’ mapping eases use; however, specificity is lost, and cases requiring more precision need manual revision.

This resource enables lifelong disease trajectory analysis, transgenerational studies and longitudinal comparison of clinical practices and treatments. Analysing medical history over long periods of time is essential to understanding the development and long-term effects of complex diseases, disease patterns and population trends. Further, this mapping can easily be integrated into disease ontologies, in addition to its use in data harmonization.

In conclusion, we here make publicly available a many-to-one, unidirectional, ICD-8 to ICD-10 crosswalk which has been exceptionally useful in our research. It has allowed us to analyse data from 1969 to the present, and creating valuable results, some of which have direct clinical application as clinical decision support. To expand usability from the Danish to the international context, we supplement the Danish crosswalk with an international computerized-cum-manual conversion, which may be useful as a model for future data harmonization work.

### Supplementary Information

Below is the link to the electronic supplementary material.

Supplementary file1 (TXT 793 KB)

Supplementary file2 (PDF 148 KB)
