# Supplementary material for: A unidirectional mapping of ICD-8 to ICD-10 codes, for harmonized longitudinal analysis of diseases
Source: Eur J Epidemiol. 2023 Aug 9;38(10):1043–52. doi: 10.1007/s10654-023-01027-y (PMC10570238; doi:10.1007/s10654-023-01027-y)
Supplement: Supplementary file 2 — Supplementary file2 (PDF 148 KB) [file 10654_2023_1027_MOESM2_ESM.pdf]

Supplementary material

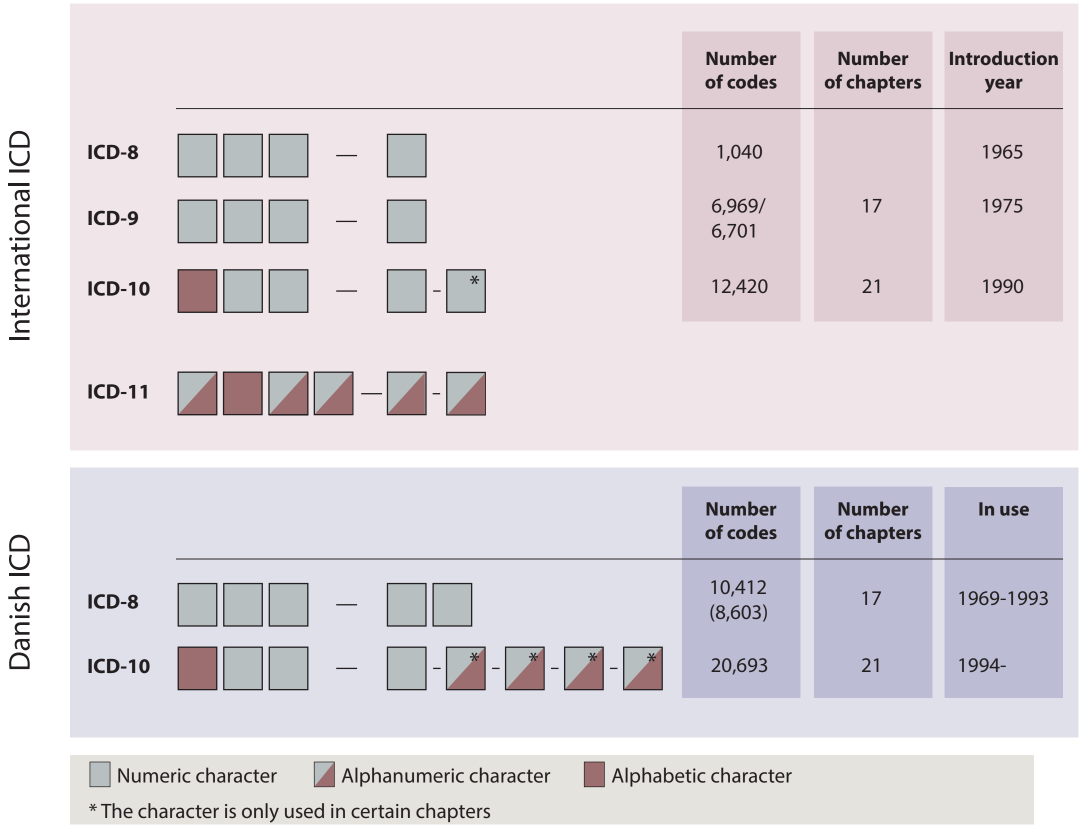

Supplementary Fig 1 | The composition of ICD codes. Overall code structure of ICD-8, ICD-9, ICD-10 and ICD-11 for the general ICD versions defined by WHO and the Danish expansions. Colours indicate the type of character (numeric, alphanumeric or letter). Information on the revisions is presented on the right. The international ICD-10 has an additional chapter XXII '*Codes for special purposes'* including temporary codes. In addition to the 26 main chapters ICD-11 contains a chapter of supplementary section for functioning assessment and a chapter of extension codes. The number of codes in the Danish ICD-8 revision includes 1,568 E-codes and 241 Y-codes.

Supplementary table 1 | Unmatched ICD-8 codes. Seven Danish ICD-8 codes have no ICD-10 match. Homosexuality is not listed as a disease or disease related condition and is excluded from ICD by WHO. The mortality codes are no longer part of the morbidity chapter.

Danish ICD-8 code	Code title in Latin
**30209**	Homosexualitas
**67709**	Mors subita e causa ignota post partum
**79580**	Mors subita infantum
**79599**	Mors subita, non violenta, causa ignota
**79629**	Mortuus admissus (inventus), causa ignota
**79639**	Mortuus sine signo morbi
**79699**	Causae aliae morbi et mortis male def.ignot.et non specif.

Supplementary table 2 | Description and example of flags characterising ICD-8 ICD-10 matches.

**Flag (n=3,271)**	**Flag description**	**Mapping example**	**ICD-8 title in Latin and ICD-10 title in Danish**	**English translation**
A	ICD-8 and ICD-10 subdivide in incomparable different directions. We match at the most detailed level ICD-8 and ICD-10 have in common. This match loses information and the ICD-10 match lesser detailed than the ICD-8 code.	805.29<br>S32	Fractura ossis sacri et coccygis<br>Fraktur af lumbale rygsøjle og bækkenet	Fracture of sacrum and coccyx<br>Fracture of lumbar spine and pelvis
B	ICD-8 and ICD-10 describe or name codes differently, however, we consider the codes similar.	241.09<br>E01.1	Struma nodosa non toxica endemic<br>Endemiske knudestruma forårsaget af jodmangel	Endemic non toxic nodular goiter<br>Endemic iodine-deficiency multinodular goiter
C	ICD-8 and ICD-10 specify according to different matters (like flag-A). In this case we find no good closest-less-detailed match, instead, we compromise on the information given in ICD-8 and do a best-possible match.	731.03<br>M71.9A	Bursitis acuta<br>Bursitis UNS	Acute bursitis<br>Bursitis unspecified
D	The matched ICD-8 and ICD-10 codes do not share the exact same incl. and/or excl. criteria. Despite different criteria, the match is still the best-possible one.	307.02<br>H52.1	Myopia<br>Myopi (eksl. degenerative myopi)	Myopic<br>Myopic (excl. degenerative myopic)
E	This flag indicates that ICD-8 and ICD-10 do not share the same level of details. ICD-8 and ICD-10 do not classify in different directions (covered by flag-A) and the difference is neither caused by incl. and/or excl. (covered by flag-D). This flag is mainly used when mapping from a more specific code to a lesser specific one, the opposite may also be done.	845.05<br>S93.6	Distorsio pedis<br>Distorsion af anden eller uspecificeret del af foden	Distorsion of the foot<br>Distorsion of another or unspecified part of the foot
F	ICD-10 splits an ICD-8 code into separate codes placed in different areas. A closest-less-detailed match is impossible and the match is done according to prevalence of the separate ICD-10 codes*. In few matches the flag is used even though a closest-less-detailed match is possible: in the case of extremely skewed prevalence where too many details will be lost if the match is done to a more general level (flag-A).	737.99<br>M20.1	Hallux valgus et varus (acquisita)<br>Erhvervet hallux valgus#	Hallux valgus and varus (acquired)<br>Acquired hallux valgus
G	ICD-8 and ICD-10 subdivide in incomparable different directions, matching to a lower common level is possible (like flag-A). Still, the contexts of ICD-8 and ICD-10 make this a better match based on medical knowledge.	851.99<br>S06.9	Contusio, dilaceratio, conquassatio cerebri<br>Intrakraniel læsion UNS	Brain contusion, dilacerations, crushing<br>Intracranial injury unspecified

*The frequency is based on an 1996-2012 LPR extraction (n = 6.2 million). #The frequency of: (1) M20.1 Acquired hallux valgus: 141,405, (2) M20.3A Hallux varus: 57. ICD, International Classification of Diseases.

Supplementary table 3 | Technical description of the mapping table. Fields are strings of variable lengths and delimited by tabs. Headings are listed in the first line of the mapping document.

Column	Field description	Character description	Mandatory
1	Danish ICD-8 code	Five digits	Yes
2	Danish ICD-8 code title in Latin	Unlimited number of characters	Yes
3	Danish ICD-10 match	Three-XX digits	Yes
4	Score	One digit (1,2,3 or 5)	Yes
5	Flag	One letter (A, B, C, D, E, F or G)	No
6	Start date of the Danish ICD-8 code	Eight digits (YYYYMMDD)	Yes
7	Last date of the Danish ICD-8 code	Eight digits (YYYYMMDD)	Yes
